# A combined three-dimensional *in vitro–in silico* approach to modelling bubble dynamics in decompression sickness

**DOI:** 10.1098/rsif.2017.0653

**Published:** 2017-12-20

**Authors:** C. Walsh, E. Stride, U. Cheema, N. Ovenden

**Affiliations:** 1 Centre for Advanced Biomedical Imaging (CABI), University College of London, Paul O'Gorman Building, 72 Huntley Street, London, UK; 2 Centre for Tissue and Cell Research, University College of London, Royal National Orthopeadic Hospital, London, UK; 3 Department of Mathematics, University College of London, Gower Street, London, UK; 4 Institute of Biomedical Engineering, Old Road Campus Research Building, University of Oxford, Oxford, UK

**Keywords:** decompression sickness, bubbles, *in vitro*, the bends, coalescence

## Abstract

The growth of bubbles within the body is widely believed to be the cause of decompression sickness (DCS). Dive computer algorithms that aim to prevent DCS by mathematically modelling bubble dynamics and tissue gas kinetics are challenging to validate. This is due to lack of understanding regarding the mechanism(s) leading from bubble formation to DCS. In this work, a biomimetic *in vitro* tissue phantom and a three-dimensional computational model, comprising a hyperelastic strain-energy density function to model tissue elasticity, were combined to investigate key areas of bubble dynamics. A sensitivity analysis indicated that the diffusion coefficient was the most influential material parameter. Comparison of computational and experimental data revealed the bubble surface's diffusion coefficient to be 30 times smaller than that in the bulk tissue and dependent on the bubble's surface area. The initial size, size distribution and proximity of bubbles within the tissue phantom were also shown to influence their subsequent dynamics highlighting the importance of modelling bubble nucleation and bubble–bubble interactions in order to develop more accurate dive algorithms.

## 1. Introduction

A reduction in ambient pressure leading to bubble formation is a process that is ubiquitous in nature and widely exploited in industrial processes [1,2]. Since the concentration of dissolved gas in a liquid is proportional to the ambient pressure, when the pressure is reduced, gas is forced out of solution and, under energetically favourable conditions, may form bubbles. Unfortunately, this process can also have deleterious effects. In marine mammals and human SCUBA (self-contained underwater breathing apparatus) divers, the accumulation of dissolved gas in tissues while at depth and subsequent ascent can lead to bubble formation within the body and ultimately to decompression sickness (DCS). The mechanisms through which bubble formation causes DCS [3] are highly contentious [4]. Evidence for inflammatory mechanisms [5,6] and direct biomechanical insults [3,7] have been proposed but there remains little consensus.

Given this lack of a clear biological mechanism, current approaches for treating and preventing DCS are based on the physical mechanisms of bubble formation and growth. Dive algorithms, which calculate safe ascent rates, are typically comprising a mechanistic computational model of tissue gas kinetics and/or bubble dynamics and a probabilistic DCS risk estimation model [8]. Despite the wide use of such algorithms, every year divers still suffer acute DCS [9] and may experience delayed effects even after treatment [10]. There is also emerging evidence that repeated but asymptomatic dives result in long-term health damage [10,11]. Consequently, there is broad interest from the commercial, military and recreational diving industries to improve and develop these algorithms. To do so, however, involves multiple, interrelated challenges. These include accurate model parametrization, experimental validation and identification of the relevant biological mechanism(s). Currently, in order for a dive algorithm to prescribe a ‘safe ascent rate’, an assumption linking the mechanistic (tissue gas kinetic and bubble dynamics) model output to the probabilistic model is needed, e.g. that the probability of DCS is a function of the total volume of bubbles or the tissue supersaturation level. A maximum-likelihood method is then used to optimize the mechanistic model parameters using a database of dive profiles and corresponding DCS incidence [12]. If the optimized parameter values fall within a physiologically plausible range, the mechanistic and probabilistic models are assumed to be valid. Given that the mechanism(s) by which bubbles cause DCS are unknown, the choice of mechanistic model output is not a straightforward decision. If a model's optimized parameter values are far outside physiological limits, it cannot be known with certainty whether the mechanistic model is incorrect or the probabilistic function has been formulated on an inappropriate mechanistic model output. Similarly, even when optimized parameter values fall within physiological limits, this does not guarantee the validity of the mechanistic model.

The most robust validation of the mechanistic model would be real-time measurements of bubble dynamics *in vivo*. Currently, the gold standard for measurement of bubbles *in vivo* is either non-invasive Doppler/transthoracic ultrasound [13] or invasive light microscopy in animal models [14]. The first technique is routinely used in human divers and provides a measure of ‘bubble severity’ post dive based on one of several scales [15]. While useful for estimating bubble quantities, these scales do not provide a good indication of the likelihood of DCS onset [16]. In addition, mechanistic models predominantly consider the extravascular bubble population which is not accurately measurable by the currently used ultrasound techniques [17]. Direct light microscopy in animal models has provided predominantly qualitative data, and often only at a single time point [14,18,19]. Microscopy of transparent animals overcomes the single time point problem; however, movement of the animals often prevents real-time bubble tracking [20,21]. *Ex vivo* models allow for direct observation by light microscopy and, to date, the works of Papadopoulou *et al*. [22] and Arieli [23] have provided important contributions regarding preferential bubble nucleation sites and multi-bubble growth dynamics from the surface of various tissue types (including fatty, aqueous and large vessel lining). These data, while useful, cannot be used to investigate the population of bubbles nucleating within tissues, or validate models which describe them. Validating mechanistic bubble models using *in vivo* or *ex vivo* models is challenging due to the large numbers of variables within *in vivo* systems and the comparative simplicity of the physical science based computational bubble models. An approach that has produced a large proportion of quantitative data suitable for computational model validation is *in vitro* models. Yount and colleagues [24–27] made extensive use of gelatin models to investigate bubble nucleation and used their data to develop and validate the varying permeability model (VPM)–a commercial diving algorithm. Van Liew *et al*. [28] used bubbles in saline to compare bubble growth to computational models. Wang *et al*. [29] also used a two-dimensional *in vitro* cell culture model to examine the decompression stresses caused by increased partial pressure of oxygen; these findings were supported by recent three-dimensional *in vitro* experimental investigations [30]. However, there remains a need for a controllable system in which bubble nucleation, growth and biological responses within tissues can be quantified in real time, as material, dive and biological parameters are systematically altered.

**Figure 1.**
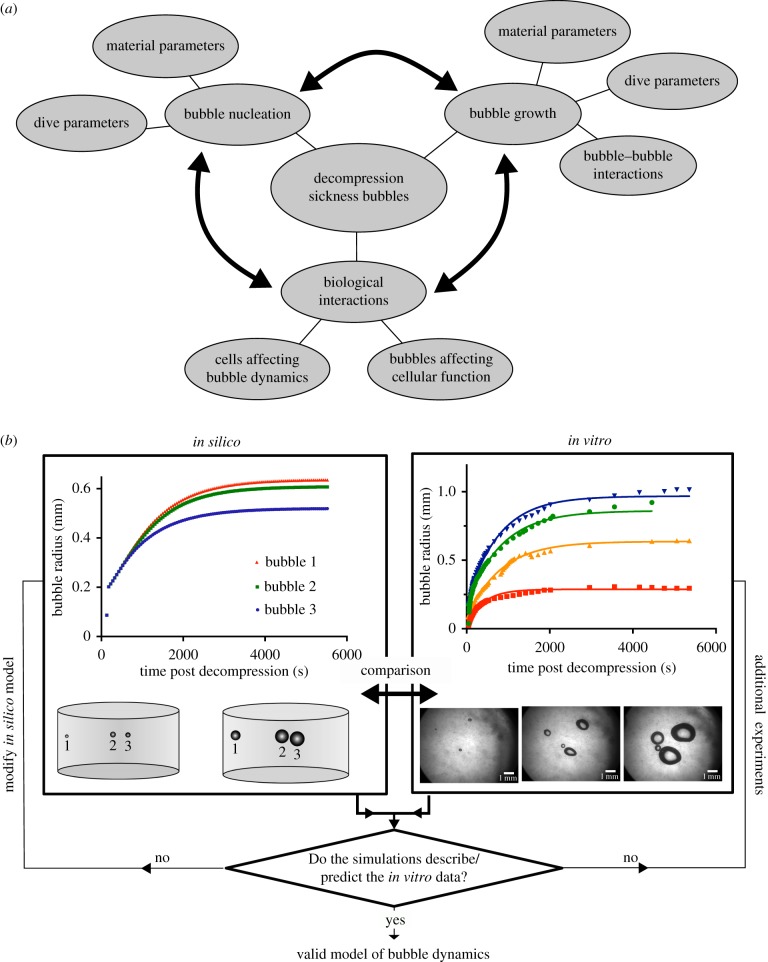
(*a*) A diagram of the various aspects of bubble dynamics that are considered relevant to DCS. (*b*) Diagram showing the methodological principles behind the use of a complementary *in silico–in vitro* approach. Comparative analyses are made via validation metrics chosen depending on the experiment. For example, for decompression experiments depicted here, the validation metrics used are the plateau radius of the bubble and the half-life of bubble growth (see the electronic supplementary material for more information). Comparison of the computational and experimental cases may be made by comparison of these metrics using various statistical tests including the extra sum-of-squares *F*-test [30]. Additional factors such as the non-spherical bubble shapes seen in the experimental case may also inform further computational development. (Online version in colour.)

The present work details the development and application of an *in silico* mechanistic model and a complementary biomimetic *in vitro* model, to investigate several areas of extravascular bubble dynamics (figure 1*a*). The *in silico* model was formulated as a three-dimensional finite difference simulation of the *in vitro* model, a type I collagen gel. Type I collagen is the most abundant extracellular matrix protein in the body [31], and such gels are widely used in tissue engineering applications, the inclusion of additional matrix proteins and engineered control of their material properties is well established [32].

A diagrammatic representation of the combined *in vitro*–*in silico* approach is shown in figure 1*b*.

## 2. Mathematical and computational methodology

### 2.1. Mathematical formulation

The *in silico* model developed in this work simulates bubble dynamics in collagen gel tissue phantoms in response to changes in external pressure. The modelling assumptions and derivation of the governing equations are described below.

#### 2.1.1. Gas transport

The transport of gas through the tissue phantom and across the bubble surface is assumed to occur by diffusion only, with all gases obeying perfect gas laws. Thus, *C^g^*, the concentration of the *g*th gas, is governed by the equation2.1
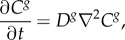
where *D^g^* is the diffusion coefficient and *t* is the time. Perfusion is not modelled to reflect the current experimental system. At the tissue phantom and bubble boundaries any gas is assumed to be dissolved in the tissue phantom in accordance with Henry's Law:2.2
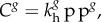
where 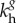 is Henry's constant and pp*^g^* is the partial pressure of the *g*th gas. At the bubble-tissue interface, the change in mass (*m*) of gas in the bubble is governed by Fick's first law:2.3
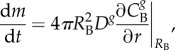
where *R*_B_ is the bubble radius and 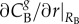 is the concentration gradient at the bubble boundary. The concentration of gas on the inner surface of the bubble (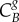) is calculated by Henry's Law once again, but with the use of 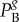, the partial pressure inside the bubble for each gas. *P*_B_ is calculated by the Young–Laplace equation:2.4
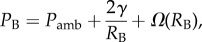
where *P*_amb_ is the ambient pressure, *γ* is the surface tension and *Ω*(*R*_B_) describes the pressure exerted by the surrounding tissue as a result of its deformation. Equations (2.2)–(2.4) can be used to derive an expression for the radius change of a bubble with time:2.5
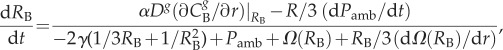
where *α* is the specific gas constant.

#### 2.1.2. Tissue elasticity

The tissue elasticity expression *Ω*(*R*_B_) introduced above was originally conceived to describe a tissue deformation threshold that, when exceeded, would lead to the distention of nerve endings, causing the joint pain characteristic of DCS [33]. Four different forms of the expression (three from the literature and one newly derived here) are now described and then compared below using a simple model of bubble growth that neglects mass transfer.

*The Bulk Modulus form* [34]2.6
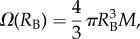
where *M* = *K*/*V*_aff_, *K* is the tissue bulk modulus and *V*_aff_ is the affected tissue volume, which is the most widely applied tissue elasticity form in the literature, appearing in several bubble models [35–37]. It has also been incorporated into the commercial Bubble Volume Model (BVM) [38], a probabilistic model that bases the risk of DCS on the total bubble volume. Following optimization based on DCS incidence, the optimized value of *K* in the BVM lay far outside the physiological range by several orders of magnitude. It was suggested by the authors that equation (2.6) captures the effects of several mechanisms restricting bubble growth and *M* should, therefore, be treated as an empirical parameter [38]. However, it has been argued [39] that the unphysiological values indicate that the tissue elasticity model of BVM is simply invalid. It is also argued [40] that the use of bubble volume as a predictor of DCS risk may be inappropriate.

*The Continuity of Displacement form* [39,41] was derived in response to concerns regarding the mathematical validity of the Bulk Modulus form. It is based on a classical linear elasticity approach to the problem, and uses the continuity of displacement at the gas liquid interface to provide a necessary boundary condition to close the model. The following expression of the Continuity of Displacement form is valid for 0 < *μ* << *K* [41], where *μ* is the small shear modulus and *K* the bulk modulus, and used in our comparison below:2.7
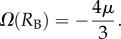
The validity of the displacement boundary condition used in the derivation is somewhat disputed [42], as displacement of a gas, unlike a solid, does not appear to have a clear physical meaning. In addition, the persistent negative value of *Ω* seems hard to justify realistically and leads to some unintuitive predictions of bubble growth.

Faced with these widely adopted, but disputed, expressions for the elastic response of the surrounding tissue, we decided to investigated their validity by comparing them to a linear elastic model of a bubble in an external medium where the problematic displacement boundary condition at the bubble surface is avoided by using the concept of a reference configuration, a common approach in continuum mechanics (see the electronic supplementary material for full derivation). The resulting expression is given by2.8
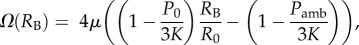
where *P*_0_ is the initial external pressure, *R*_B_ and *R*_0_ are the current and initial bubble radii, respectively. This *Reference Configuration* approach is mathematically consistent and leads to an intuitive response. This is because an initial drop in ambient pressure leads to a reduction in *Ω*(*R*_B_), thus encouraging bubble growth, but any growth in bubble radius is then positively resisted by the tissue. Our analysis, however, provides no justification for either the Bulk Modulus or Continuity of Displacement forms and a decision was made not to adopt either in our simulations. Instead, we chose to examine a third formulation originally derived to predict void formation in rubber [43].

*The Hyperelastic form* was first applied to DCS to investigate the effect of tissue elasticity on bubble nucleation [44]. The form is derived from a hyperelastic strain-energy density function and can be written2.9
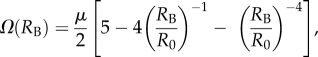
where *μ* is the small shear modulus.

**Figure 2.**
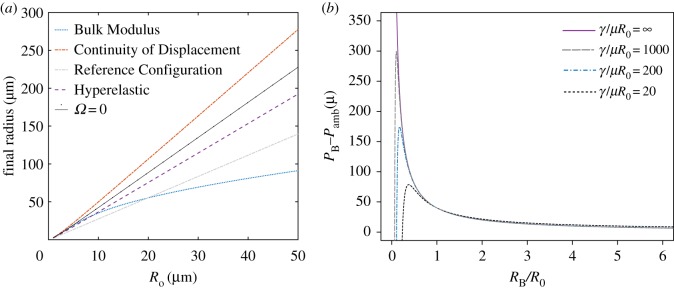
(*a*) Comparison of the four forms (and *Ω* = 0) of the tissue elasticity expression. Initial and final bubble radii are shown for a decompression with no mass transfer from 10^6^ to 10^4^ Pa (other pressure changes, not shown here show a similar trend). *M* = 5 × 10^−2^ Pa µm^−3^ [38], *K* = 2.2 GPa [45], *γ* and *μ* taken from table 1. (*b*) The Laplace equation using the hyperelastic term for variations of the non-dimensional parameter *γ*/*μR*_0_.

Figure 2*a* shows how adopting the four different forms of *Ω*(*R*_B_) affects the predicted final radius (*R*_B_) versus initial radius (*R*_0_) of a bubble subjected to a pressure reduction from 10^6^ to 10^4^ Pa. The parameter values chosen for these computations are typical of those used in previous DCS solutions in the literature; the solution for *Ω*(*R*_B_) = 0 (no tissue response) is also plotted. No mass transfer is assumed in these computations and hence the change in radius is determined simply by Boyle's Law (with constant temperature assumed). As one can observe, larger differences occur between the expressions at larger initial bubble sizes where surface tension is less dominant. Adopting the Bulk Modulus term appears to restrict the growth of larger bubbles significantly more than the other expressions, whereas adopting the Continuity of Displacement form leads to the opposite extreme of much higher final radii due to its persistent negative sign.

**Table 1.** Parameter values used in the *in silico* model. Individual tables for each parameter can be found in the electronic supplementary material.

parameter	description	units	value
*δt*	time step	s	variable (dependent on stability requirements)
*h*	spatial grid step	m	variable (dependent on stability requirements)
*L_x,_L_y,_L_z_*	size of tissue phantom	m	variable (dependent on experiment)
*γ*	surface tension	N m^−1^	0.042–0.07
*μ*	shear modulus	Pa	0–4 × 10^6^
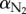	specific gas const.	kJ kg^−1^	92.07
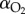	specific gas const.	kJ kg^−1^	80.6
LN_2_	Otswald's const. *N*_2_	—	0.014
LO_2_	Otswald's const. *O*_2_	—	0.013–0.027
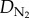	diffusion coeff.	m^2^ s^−1^	1 × 10^−10^–2.7 × 10^−9^
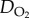	diffusion coeff.	cm^2^ s^−1^	1 × 10^−10^–2.7 × 10^−9^
*R*_min_	minimum radius	m	*h*
*m*_f_	mole fraction	—	0.2 : 0.8 O_2_ : N_2_

From our elasticity analysis and the comparison in figure 2*a*, the Hyperelastic form was chosen for all subsequent simulations in this paper as it has strong foundation in the literature with validation for bubble growth and application to healthy tissue biomechanics [43,46]. In addition, it is easy to compute and fully nonlinear enabling strain-stiffening behaviour to be incorporated which may be important for larger strains with significant bubble interactions. The Reference Configuration form, however, seems applicable in many cases of isolated bubble growth, and its assumptions are likely to remain valid when considering growth of bubbles smaller than the tissue scale. However, with the limited knowledge of bubble size distributions and the likelihood of bubble interactions, a mathematically consistent nonlinear model was preferred for this first attempt at validation and model development.

Figure 2*b* shows the plot of the Laplace equation with the Hyperelastic form for different values of the dimensionless parameter 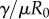. For smaller values of 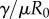, resistance to bubble growth is predominantly due to tissue elasticity, whereas for larger values the resistance is predominantly due to surface tension.

#### 2.1.3. Non-dimensionalization

Having chosen the form of the tissue elasticity expression, the change in bubble radius with time can now be derived for O_2_ and N_2_ in non-dimensional form as2.10
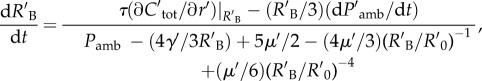
where *C*_tot_ is the total dissolved gas concentration, *τ* is a combined parameter and the prime (′) is used to denote non-dimensional variables:
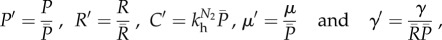

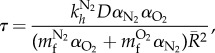
where 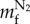 and 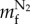 are the mole fractions of N_2_ and O_2_ respectively.

#### 2.1.4. Nucleation

Nucleation is a complex phenomenon and a formidable modelling problem in its own right. Whether nucleation occurs is dependent upon (i) the sum of the volume energy (associated with the formation of a volume of a new phase), which reduces the overall free energy, and (ii) the surface energy (associated with phase boundary creation), which increases free energy. These two opposing terms lead to an energy barrier to nucleation depending on the radius of the nucleus [3].2.11
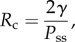
where *R*_c_ is known as the critical radius and *P*_ss_ is the tissue supersaturation, i.e. the difference between a tissue's dissolved gas concentration and the equilibrium concentration given the external pressure. The critical radius may vary due to the presence of contaminants or surfaces within the liquid phase which alter the surface energy. This is known as heterogeneous nucleation and is broadly accepted to be the form of nucleation occurring in DCS [3].

In this initial computational implementation, nucleation is not modelled explicitly; instead the simulated tissue phantom is assumed to contain a defined initial population of bubbles. These bubbles are randomly distributed within the phantom and set to an initial minimum size (*R*_0_ *=* computational spatial grid size)*.* At this minimum size nuclei are prevented from shrinkage by the imposition of a no-flux boundary condition on the bubble surface. Once decompression commences, bubbles may grow according to Boyle's Law and once *R*_B_ > *R*_0_ mass flux can occur. This is similar, in principle, to Yount *et al*.'s variable permeability model in which a skin of hydrophobic molecules is assumed to stabilize micronuclei against collapse by rendering the bubbles gas impermeable [24].

### 2.2. Computational implementation

To numerically implement the governing equation (2.10) and equation (2.1), the tissue phantom is defined as a three-dimensional array of nodes with distance 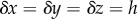 between each node. Each node is described by its three-dimensional Cartesian coordinate and represents a unit volume of the system. At each node, the concentration of dissolved gas and phase (liquid or gas) of the node is stored. The pressure profile is discretized into time portions 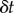 and, at each time point, the pressure external to the tissue block *P*_amb_ is given by this profile. *P*_amb_ is used in Henry's Law (equation (2.2)) to set the dissolved gas boundary values at the tissue phantom edge. From these boundary values, the dissolved gas concentration at every liquid node can be calculated. These values are used to calculate the dissolved gas gradient at the tissue phantom bubble interface which is used in equation (2.10) to calculate the new radius of the bubble. A more detailed description of the computational implementation is given in the electronic supplementary material.

### 2.3. Parametrization

The parameters used in the model are listed in table 1 and in the electronic supplementary material. The numerical parameters (*δt*, *h*) were chosen for each simulation to ensure numerical efficiency and convergence of the solution. The material parameters (*D*, *μ*, *γ*, 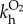
and 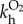) were defined based on a review of the relevant literature, which sought to set limits for the case of collagen gels and tissues (see the electronic supplementary material for more details).

#### 2.3.1. Sensitivity analysis

**Figure 3.**
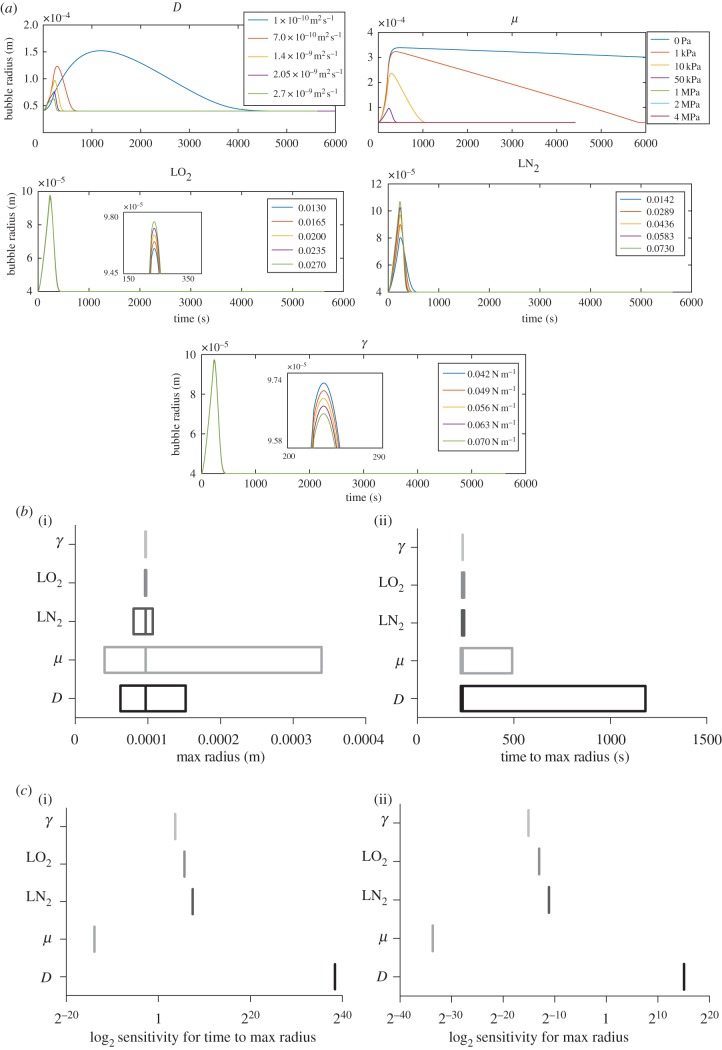
(*a*) Radial time course for the sensitivity analysis of each of the five material parameters. In all cases, the fixed parameter values are the modal values of the range (*γ* *=* 0.056 N m^−1^, LN_2_ *=* 0.0436, LO_2_ *=* 0.02, *μ =* 2 × 10^6^ Pa, *D* *=* 1.4 × 10^−9^ m^2^ s^−1^), a single bubble *R*_0_ *=* 0.04 mm was centred in a grid of 1.28 mm^3^. Pressure profile was a decompression from 30 m in 3.75 min (ascent rate approx. 0.133 m s^−1^), tissue was assumed to be fully saturated at the start of decompression. (*b*) The change in maximum radius (i) and time to maximum radius (ii) over the full range of model parameter values. (*c*) The sensitivity (Δ bubble metric/Δ parameter value) of the maximum radius (i) and time to max radius (ii) to these parameters.

The sensitivity of the maximum bubble radius and its derivative to changes in the different material parameters was computationally determined. Simulations were conducted on a 1.28 mm^3^ grid with *h* *=* *R*_0_ = 0.04 mm (mean experimentally measured *R*_0_ in cases of nucleation). A single bubble located at the centre of the grid was exposed to a decompression corresponding to a depth change of 30 m in 3.75 min (ascent rate approx. 0.133 m s^−1^). The tissue was assumed to be saturated with air at the start of the decompression, hence time *t* = 0 represents the beginning of the ascent from a saturation dive. Figure 3 shows the results of the simulations for all five material parameters. From figure 3*a*, it can be seen that increases in the parameters *D*, *μ* and *γ* led to a decrease in the maximum bubble radius (*R*_max_) and a decrease in the time taken to reach this maximum (*t*_max_). Increases in both LN_2_ and LO_2_ led to an increase in *R*_max_ but a decrease in *t*_max_. In the context of tissue phantoms or human tissue these results suggest that stiffer phantoms will resist bubble growth to a greater degree; smaller diffusion coefficients, i.e. denser tissue will contain larger bubbles that persist for a longer time; tissues with lower surface tension will contain bubbles with a larger radius; finally, more lipid rich tissues (higher solubility coefficient) will also tend to contain larger bubbles. Support for these conclusions is found widely in the modelling literature [34,40,47,48] and to a certain degree in the experimental literature [22,28,49]. However, for the majority of data from *in vivo* experiments, it is difficult or even impossible to separate the individual effects of material parameters from each other and also from those of perfusion.

The sensitivities of bubble size and growth rate to the model parameters are shown in figure 3*b*. The top panel shows the range of *R*_max_ (left) and *t*_max_ (right), over each parameter's physiological range. From this top panel it can be seen that *μ* has the largest effect upon *R*_max_, while *D* has the largest effect upon *t*_max_. The lower panel shows the sensitivity of *R*_max_ and *t*_max_ to each parameter. These results show that *R*_max_ and *t*_max_ are relatively insensitive to *μ* and that its large impact is due to its wide physiological range. LN_2_, LO_2_ and *γ* all have a similar influence that is larger than that of *μ* but the parameter to which *R*_max_ and *t*_max_ are most sensitive is *D.* This finding is not unexpected, as *D* plays a role both in the transport of gas through the bulk tissue equation (2.1), and also in the diffusion across the bubble-tissue interface equation (2.10). Hereafter these will be referred to as *D*_bulk_ and *D*_surf_, respectively. Thus, it was decided that the most appropriate way to validate the *in silico* model would be through experimental variation of *D*.

It is important to note that while *γ* appears to have a relatively small influence, the initial radius of the bubble will affect the magnitude of the surface tension force and thus the sensitivity of bubble radii to *γ* will vary with *R*_0_. A more appropriate way to consider the sensitivity to surface tension is by the radius at which the surface tension force substantially contributes to the bubble's internal pressure. For the lowest and highest values of surface tension used in these simulations (*γ* = 0.04 and *γ* = 0.073 N m^−1^) the surface tension contribution to the total bubble pressure is only 2% and 3.5%, respectively, at the initial radius of 0.04 mm. If an initial radius of 0.002 mm was used the proportions would be 27% and 36%, respectively. It is, therefore, important not to discount the importance of surface tension until the initial bubble size distribution has been established.

## 3. Experimental methods

### 3.1. Pressure chamber and image analysis

Experiments were performed using the experimental system described previously [30]. This consists of a microscope-mounted pressure chamber which can hold a single collagen gel. Optical windows in the upper and lower faces of the chamber allow imaging of bubbles in the gel in real time. Bubble radii were extracted using the semi-automated image analysis method described in the electronic supplementary material and in a previous study [30].

### 3.2. Collagen gel fabrication and plastic compression

Collagen hydrogels (0.5 ml) consisting of 0.4 ml monomeric collagen (rat tail collagen type I (First Link, UK)), 0.05 ml of 10× Modified Eagle's Medium (Gibco, UK) were neutralized through the drop-wise addition of 5 M NaOH (Sigma, UK) then stored on ice for 1 h. After 1 h, 0.05 ml of phosphate buffered saline (Oxoid, Thermo Scientific, Loughborough, UK) was added. This final gel solution was pipetted into individual wells of a 48-well plate and incubated at 37°C for 15 min, these are termed hydrogels. In some cases gels were plastically compressed [32] altering their collagen : water ratio and hence decreasing their diffusion coefficient [50] and increasing their elastic moduli [51].

Plastic compression was achieved by placing two absorbent paper discs (10.5 mm diameter) on top of the gel and a cylindrical roll of paper (diameter 10.5 mm) on top of these (Whatman grade I) for 45 s. These compressed gels are hereafter referred to as dense gels. Finally, all gels were covered in 0.6 ml (DMEM), 2 mmol l^−1^ glutamine high glucose (Sigma, UK), with 10% fetal calf serum (First Link, UK) and penicillin streptomycin (p s^−1^) (500 unit ml^−1^ and 500 µ ml^−1^) (ICN Biochemicals, UK). They were incubated again at 37°C for 8–10 h.

## 4. Validation

### 4.1. Diffusive mass transfer validation

The aim in using a relatively short plastic compression time (45 s) was to alter the diffusion coefficient sufficiently to affect bubble dynamics while having a minimal effect upon the other material parameters. The change in proportion of collagen between the hydrogel and the dense gel was 0.53% ± 0.15% (by weight increase of plunger) [52,53]. Based on previous measurements this was expected to reduce the diffusion coefficient by a maximum of 1 × 10^−9^ m^2^ s^−1^ [50]. The plastic compression should not have produced a measurable change in elastic modulus [51], and only large bubbles of radius > 0.09 mm were selected to minimize the effect of changes in surface tension (bubble population radial range 0.09–0.29 mm, mean = 0.197 mm, s.d. 0.05 mm). Bubbles of radii smaller than approximately 0.09 mm showed a markedly different oscillatory pattern with such bubbles often dissolving entirely during the compression phase of the cycle, indicating the dominance of the surface tension force in such cases. Given the low sensitivity of bubble growth to LN_2_ and LO_2_, it was assumed that the variation in the diffusion coefficient would be the dominant cause of variations in bubble dynamics. Initial bubble radius and bubble spacing were controlled by introducing bubbles via gentle agitation of the ungelled collagen with a pipette tip. This method avoided the need for spontaneous bubble nucleation and enabled accurate measurement of initial bubble radii for comparison with the computational results. Two sample groups, hydrogels and dense gels, were subjected to an oscillatory pressure profile (0–1.38 × 10^5^ Pa (0–20 psi) over 30 s for five cycles) to enable comparison with the computational results. This pressure profile reduced the possibility of bubble–bubble interactions that were not accounted for in the initial *in silico* model.

**Figure 4.**
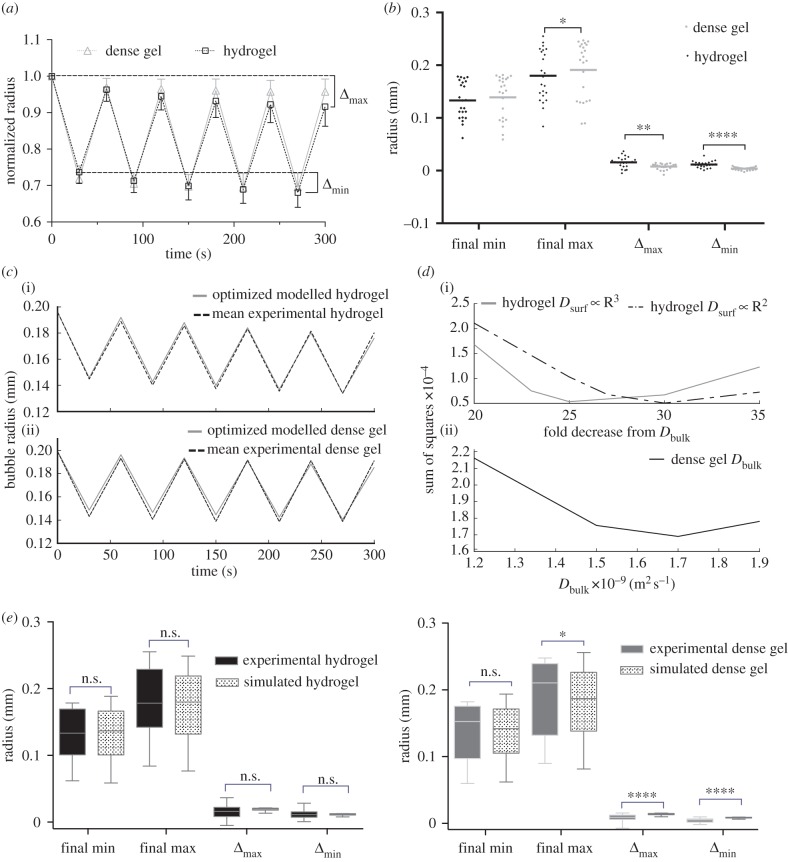
(*a*) Normalized bubble radii over the time course in which the external pressure was varied between 0 and 20 psi over 30 s for five consecutive oscillations, sequential maximum and minimum radii were measured manually in ImageJ (*n* = 23). The maximum and minimum radius changes (as marked in the graph) as well as the final minimum and maximum radii were used in the subsequent statistical analysis. (*b*) Comparison of bubble dynamics in the experimental hydrogel and dense gel, significance level *p* = 0.05 for paired *t*-test. (*c*) Overlay of the fitted models to the mean experimental hydrogel (i) and dense gel (ii) *R*_0_ = 0.2 mm (mean experimental *R*_0_ in oscillatory pressure experiment). *L_x_* *=* *L_y_* *=* *L_z_* *=* 1.28 mm, *h* *=* 2 × 10^−5^ m, LN_2_ = 0.145, LO_2_ = 0.027, *μ* = 40 Pa and *γ* = 0.07 N m^−1^. (*d*) Fitting via minimization of the sum of squares for the hydrogel (i) for both fold change of *D*_surf_ from *D*_bulk_ and for 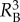 or 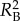 dependence of *D*_surf_. (ii) The minimized sum of squares for *D*_bulk_ in the case of the dense gel. (*e*) Comparison of simulated pressure oscillations to experimental data for all measured *R*_0_ values for hydrogel (left) and dense gel (right). Comparison of experimental and simulated data is based on the bubble metrics used in (*b*), paired *t*-test significance level *p* = 0.05 was calculated for each metrics.

Bubbles in both groups were pair-wise matched according to their initial radii |(*n* = 23 bubble pairs within five different gels per condition). Figure 4*a*,*b* shows increasing difference in radii profile's over pressure cycles which is shown to be significant via the various metrics measured. This finding confirmed the effectiveness of plastic compression as a means of varying material parameters. Simulation data were fitted to the mean bubble's radial timecourse for the hydrogel and dense gel via minimizing the sum of squares (in both cases the mean *R*_0_ = 0.2 mm). Simulations of this single mean bubble in the hydrogel case (figure 4*d*(i)) indicated that *D*_surf_ was a function of *R*_B_^2^ and had a value of *D*_bulk_/30 at *t* = 0, *R*_B_ *=* *R*_0_*.* Using the mean dense gel data and the relation between *D*_surf_ and *D*_bulk_ found for the hydrogels (i.e. 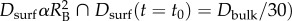, *D*_bulk_ in the dense gel was found to be 1.7 × 10^−9^ m^2^ s^−1^, (figure 4*d*(ii)) which accords with previous data [50]*.* The fitted modelled and mean experimental data are overlaid in figure 4*c* for comparison*.* The *R*_B_^2^ relationship and reduced *D*_surf_ are suggestive of contamination of the air–liquid interface by adsorbed surface active molecules [54,55] many of which are likely to be present in both the *in vivo* case and in our *in vitro* tissue phantom.

Using these *D* values, further simulations were conducted for each experimental (bubble pair) *R*_0_. The simulated and experimental data are compared in figure 4*e*. No significant differences were measured between simulation and experiment for the hydrogel. For the denser gel, however, simulations consistently predicted a greater reduction in bubble radius over successive oscillations than that measured experimentally. This discrepancy has several potential sources all of which warrant further investigation. These include the poroviscoelastic nature and anisotropy of the denser gels; both of which are exhibited in human tissue [56].

### 4.2. Dive parameter investigation

The combined approach was next used to investigate bubble dynamics under pressure profiles more representative of dive situations. Hydrogel tissue phantoms were subjected to a pressure profile consisting of a compression (1 psi s^−1^ = 6.9 × 10^4^ Pa s^−1^), a time at the maximum depth of (130 psi = 9.0 × 10^5^ Pa) and, finally, a decompression. Time at depth and decompression rate were varied for these experiments. Bubbles were no longer introduced during the gelation period but nucleated spontaneously during decompression. For each bubble that nucleated, the radius–time curve was fitted to an exponential growth equation via nonlinear regression (see the electronic supplementary material, figure S4.) The plateau radius and half-life resulting from the regression were used as validation metrics by which to evaluate each dive parameter's effect and compare the simulation results. Means for each gel were calculated (*N* = 3 values per dive condition).

Simulations of each dive profile were undertaken using the *D*_bulk_ and *D*_surf_ values fitted previously and the fixed values for the other material parameters*.* In the simulation, three bubbles were randomly distributed within the phantom at the outset. The positions and initial radii of the simulated bubbles were the same in all simulations. The same nonlinear regression analysis was used on the simulation data.

**Figure 5.**
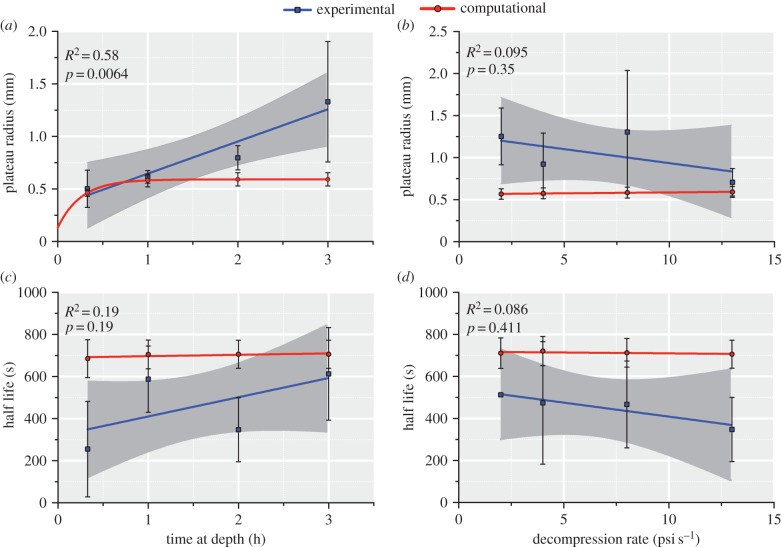
A comparison of bubble metrics from the *in silico* and *in vitro* models for dive parameter variations. In all cases, compression was at (1 psi s^−1^ = 6.9 × 10^4^ Pa s^−1^), to a maximum depth (130 psi = 9.0 × 10^5^ Pa). The mean and standard deviation are shown for data points. A linear or nonlinear regression fit is also shown with the 95% confidence intervals displayed in the experimental cases. *R*^2^ and *p*-values (*F*-test with null hypothesis that the slope is zero) are reported for the experimental case only. The fits for the experimental and computational cases were statistically compared via an extra sum-of-squares *F*-test. The simulated tissue phantom was 11 mm in diameter and 5 mm in depth with *h* *=* 8.65 × 10^−5^ m and *R*_0_ *=* *h*, LN_2_ = 0.145, LO_2_ = 0.027, *μ* = 40 Pa and *γ* = 0.07 N m^−1^. *D*_bulk_(*t* *=* *0*) *=* 2.5 × 10^−9^ m s^−2^, *D*_surf_ *=* *D*_bulk_/30. (Online version in colour.)

Figure 5 reveals a significant positive linear correlation between time at depth and the mean plateau radii of bubbles in the experimental case (*p* = 0.0064). No trend was found between decompression rate and mean plateau radii; nor between bubble half-life and either dive parameter.

Both the simulated and experimental data indicate that the surrounding dissolved gas concentration (as controlled by time at depth) has a greater impact on bubble dynamics than the initial bubble growth rate (controlled by the decompression rate).

It is interesting to note that the rate of bubble growth as indicated by the half-lives does not appear to vary with time at depth, whereas the final radial distribution does. This result coupled with the lack of trend in bubble metrics with decompression rate implies that the rate of bubble growth is not limited by the available dissolved gas but by mass transport across the bubble surface.

Within the simulated data the plateau radii were persistently lower than the experimental measurements, and half-lives consistently longer. Quantitative comparison of the experimental and simulated data was done via an extra sum-of-squares *F*-test (Prism 6 Graphpad). In the case of the plateau radius dependence on time at depth (figure 5*a*), a nonlinear fit was statistically indicated for the computational data (*p* = 0.499) whereas a linear fit was statistically indicated for the experimental case. For the case of the half-life dependence on time at depth (figure 5*b*), a linear fit was indicated for the computational data. The slope of this fit was not significantly different from the experimental data (*p* = 0.21); however, the intercepts were significantly different (*p* = 0.002). The same pattern (statistical differences between intercept values but not between slopes) was true in the decompression rate data for both plateau radius (figure 5*c*) (*p* = 0.0002 for intercept and *p* = 0.39 for slope) and half-life (figure 5*d*) (*p* = 0.0033 for intercept and *p* = 0.29 for slope).

This result suggests that while similarities may be found between simulations and experiments, there may be systematic errors in the computational model (the persistent significant difference in intercepts) and assumptions made in establishing the model may not be capturing the experimental reality.

An indication of what these systematic errors and/or flawed assumptions could be is found in the experimental data. The low *R*^2^ values across all experimental cases, even in the case of the statistically significant trend for plateau radius and time at depth (figure 5*a*), indicate variance in the experimental data which cannot be attributed to the dive parameter variation.

Variation in the number of bubbles in the samples, the position of bubbles within the gel (relative to the phantom boundaries and other bubbles) and their initial radii are all factors which could cause the low *R*^2^ values and are also factors not accounted for in the computational model. In the simulations each of these factors was held constant across all simulations and initially assigned randomly, in the case of the spatial position and number of bubbles or constrained by computational requirements for *R*_0_. These additional factors were further investigated by computational and experimental methods.

#### 4.2.1. Initial bubble radius

**Figure 6.**
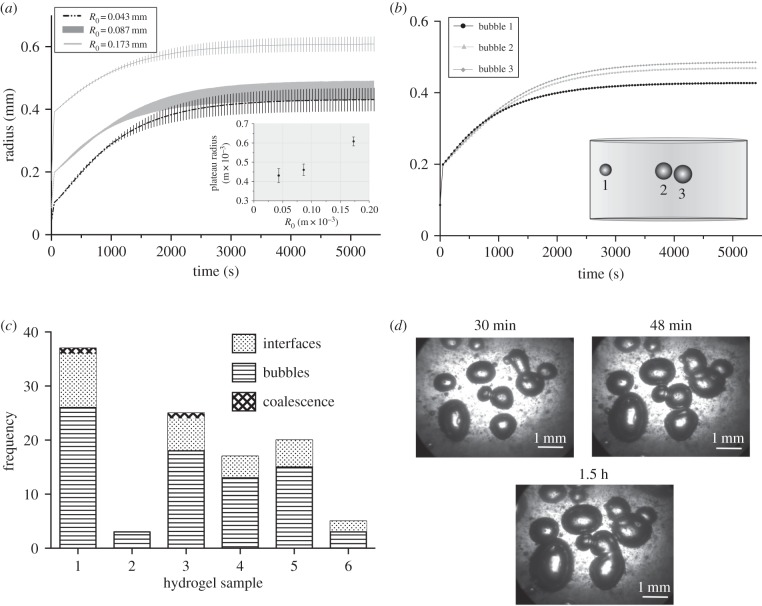
(*a*) Graph showing the relation between radial timecourse and variation in initial bubble radius *R*_0_ for decompression profile with 20 min at a depth 9.0 × 10^5^ Pa with 8.9 × 10^4^ Pa s^−1^ decompression rate and 6.9 × 10^4^ Pa s^−1^ compression rate. All other simulation parameters were the same as those in figure 5. Error bars show the range of radial values for three randomly positioned bubbles all with the same initial radius. Inset shows the relation between *R*_0_ and mean plateau radius. (*b*) Graph showing the relation between position of the bubbles within the tissue phantom and the radial time course of simulated bubbles. The inset is a diagram showing the relative positions and final radii of the three simulated bubbles. (*c*) Stacked bar chart showing the max number of bubbles, max no. of bubble–bubble interfaces formed and total number of coalescence incidences (*N* = 6 hydrogels). All samples were decompressed at 8.9 × 10^4^ Pa s^−1^ (13 psi s^−1^) after 4 h at maximum pressure 8.9 × 10^5^ Pa (130 psi). The r.h.s. shows three examples of the imaged bubbles from sample 3, where coalescence can be seen in the 1.5 h image (all times noted are as the time post decompression.).

As nucleation is a stochastic process it was not possible to experimentally vary *R*_0_ and so its effect was, therefore, investigated primarily using the computational model. The results are shown in figure 6*a*. As one observes, increasing *R*_0_ increased the plateau radius nonlinearly (figure 6*a*,inset), as expected given the larger initial surface area for gas to diffuse through and the lower surface tension. The initial radii cannot be directly controlled in the experimental system and direct measurement is likewise unfeasible. However, theoretical calculations of the initial micronuclei radial distribution may be possible. If the supersaturation of the tissue phantom can be calculated, based on the dive profile, then the critical radius (the radius above which micronuclei will grow into bubbles, rather than dissolve) may be calculated from equation (2.11).

For example, given that the tissue phantom is completely saturated during the 4 h time at depth pressure profile, the maximum supersaturation would be 9.0 × 10^5^ Pa (for the max depth used here) and hence the critical radius equals 0.15 µm. As the supersaturation will not be constant throughout the tissue but will vary both spatially and temporally, initial bubble radii would likewise be predicted to vary both spatially and temporally. Quantification of each bubble's location (both three-dimensional spatial position and temporally) could be used in conjunction with simulations of the corresponding tissue phantom supersaturation to fit the micronuclei radii distribution. Preliminary data investigating both these variables show promise and are the subject of continued investigation.

#### 4.2.2. Bubble spatial distribution

Bubble spatial distribution was also investigated computationally. Figure 6*b* shows the results for three simulated bubbles. As can be seen, the bubble nearest the edge of the phantom has the lowest plateau radius (bubble 1). Those closer to the centre exhibit a slower rate of growth initially (due to the lower dissolved gas concentration in their immediate neighbourhood); but continue to grow for longer, as the centre of the phantom takes longer to desaturate. In the experimental system, the position of the bubbles within the phantom could not be accurately measured in the *z*-(depth) plane, and, therefore, this result cannot be directly validated. However, a non-uniform bubble distribution (in the *z*-plane) was observed (see electronic supplementary material, figure S5) and has been noted in other experimental works [25].

A non-uniform bubble distribution coupled with the computational result (bubbles further from the phantom boundaries have larger plateau radii) leads to the insight that if the average bubble distance from the boundaries was greater in experiments than simulations, the mean plateau radius would be greater in the experimental data. The converse is also true.

#### 4.2.3. Bubble–bubble interactions

Bubble–bubble interactions are also likely to have contributed to experimental variability and to the discrepancies between experimental and simulated data. For many processes, such as polymer foaming or volcanic melt dynamics, bubble coalescence has a significant impact on the bubble population dynamics [57]. The likelihood that a bubble collision results in coalescence is dependent on the balance between the film drainage time and energy of deformation [58]. The faster two bubbles approach one another, the greater the likelihood of coalescence. In the *in silico* model, bubble–bubble contact was assumed to result in immediate coalescence, resulting in a single bubble with a mass equal to the sum of the previous bubbles. Coalescence frequency was investigated in the experimental system as shown in figure 6c. The number of bubbles in the samples, the maximum number of bubble–bubble interfaces that formed and the number of coalescence events that occurred were counted. It is clear from the data that coalescence was rarely the outcome of bubble–bubble contact; instead bubbles tended to deform upon contact with one another. In cases where coalescence did occur it took a considerable period of time (approx. 1 h).

#### 4.2.4. Number of bubbles

The number of bubbles that nucleate will impact on all of the above factors; this was experimentally investigated and showed a significant positive linear trend with time at depth, but no trend was found with decompression rate [19]. Attempts to increase the number of bubbles in the computational model resulted in numerous incidences of bubble–bubble contact and, therefore, coalescence was frequent and often resulted in termination of the simulation as the resultant bubble overlapped the phantom edges. Given that frequent coalescence was not seen in the experimental case of bubble–bubble contact, further work is needed to develop a more sophisticated coalescence model and to account for non-spherical bubbles.

## 5. Implications for dive algorithms

The work presented here aims to provide a means of directly validating dive algorithms and provide guidance for their further development. Our results re-enforce the notion that the Bulk Modulus form of the tissue elasticity expression is inconsistent with solid mechanics and should be viewed, in its current form, as an empirical expression only. While the hyperelastic form was adopted in our analysis, further characterization of a tissue's mechanical response to bubble growth is required.

Our results support the validity of dive algorithms using smaller diffusion coefficients for the gas–liquid interface compared to bulk tissue values such as the BVM [42]. In addition, the work shows that better modelling of bubble populations and, specifically, the interactions between bubbles within such populations is needed. The most important conclusion from the results presented here is the need to accurately characterise initial bubble size and spatial distribution. Further investigation of bubble nuclei is possible with this system and it could provide an important tool for systematically probing bubble nucleation. The VPM remains the only commercially applied model of nucleation; and while this has itself been validated in gelatin models, further work is needed to understand the influence of different tissue compositions as highlighted in other *in vivo* and *ex vivo* work [20,22,23]. One important point to note in relation to current dive algorithms is the absence of perfusion in the current model. Perfusion is an important feature in tissue gas kinetics and there are various models of perfusion incorporated into dive algorithms [8]. To extend the current computational model to include perfusion would be computationally simple; to incorporate perfusion into the experimental system, while possible would be technically difficult. In its current form, the system can be adapted to reflect different tissues in terms of their biological constituents [30], and thus the system is well suited to mimic diffusion-limited tissues, i.e. where perfusion is poor or tissue is avascular such as articulate cartilage.

## 6. Conclusion

This study has developed and used a combined experimental and computational approach to investigate bubble dynamics in tissue phantoms. Providing real-time bubble growth dynamics eliminates a hypothetical probabilistic link between the bubble population and DCS symptoms, meaning mechanistic bubble models may be directly validated.

Using this combined approach, a mathematical form of the governing equations has been developed that includes a hyperelastic elasticity term as a possible replacement for the Bulk Modulus form.

Sensitivity analysis has revealed that the diffusion coefficient is the most influential material parameter. Additionally, it has been shown that the diffusion coefficient varies between the bubble surface and the bulk tissue, with a lower value for the bubble surface and a surface area dependence. These data support the validity of dive algorithms which use lower values for *D*_surf_ and vary the value based on surface area [42].

It has been shown that bubble dynamics even in this relatively simple system are highly complex, and that many interactions may occur between bubbles which impact the population dynamics. In order to capture these population dynamics it is crucial to establish the initial bubble radii and their spatial distribution. These are not only important parameters in the growth of the individual bubbles, but also determine the likelihood that bubbles will interact with one another. Initial bubble nuclei and spatial position are predominantly determined by a nucleation model. Therefore, this work indicates that parametrizing and validating a model of nucleation is one of the most crucial research avenues in developing our understanding of, and preventing, DCS.

## Supplementary Material

Derivations, data, details of computational implementation, and additional notes for A combined 3D in vitro - in silico approach to modelling bubble dynamics in decompression sickness

## Data accessibility

The three-dimensional finite difference bubble growth code can be downloaded from Github: https://github.com/ucbpclw/Bubble-code. Details of implementation can be found in the electronic supplementary material. Raw data for figures 4 and 5 are available on figshare: https://doi.org/10.6084/m9.figshare.5644705.v1.
