# Supplementary material for: A combined three-dimensional in vitro–in silico approach to modelling bubble dynamics in decompression sickness
Source: J R Soc Interface. 2017 Dec 20;14(137):20170653. doi: 10.1098/rsif.2017.0653 (PMC5746571; doi:10.1098/rsif.2017.0653)
Supplement: Derivations, data, details of computational implementation, and additional notes for A combined 3D in vitro - in silico approach to modelling bubble dynamics in decompression sickness [file rsif20170653supp1.pdf]

A combined 3D in vitro - in silico approach to modelling bubble dynamics in decompression sickness: Supplementary Information

C. Walsh, N. Ovenden, E. Stride, U. Cheema

November 27, 2017

1 Symbols

R_B	Current radius
R_{ref}	Reference radius
U_r	Displacement
U_{rr}	Radial displacement
$U_{\sigma\sigma}$	Angular displacement
$U_{\phi\phi}$	Angular displacement
μ	Small shear modulus
σ	Stress
K	Bulk modulus
R_0	Initial radius
P_0	Initial pressure
P_{amb}	Externally applied pressure
P_B	Pressure in the bubble
γ	Surface tension
$\mathcal{R}$	Universal gas constant
T	Temperature
C_B^g	Concentration of *gth* gas in bubble
α_g	Specific gas constant
k_h^g	Henry's constant
M	Molar mass
V	Volume
V_B	Bubble volume
m_g	Mass of *gth* gas
$\Omega(R_B)$	Expression for tissue elasticity

2 Linear Elasticity derivation

The initial starting point for the derivation is the statement of infinitesimal strain theory as made by [1] where a derivation for σ_{rr} (radial component of stress) can be found in Problem 2 of [1].

$$\frac{1}{r^2}\frac{d}{dr}\left(r^2 ur\right) = const = 3a \quad (1)$$

$$U_r = ar + \frac{b}{r^2} \quad (2)$$

$$U_{rr} = \frac{\partial u_r}{\partial r} = a - \frac{2b}{r^3} \quad (3)$$

$$U_{\sigma\sigma} = a + \frac{b}{r^3} \tag{4}$$

$$\sigma_{rr} = 3Ka - \frac{4b\ mu}{r^3} \tag{5}$$

At this point boundary conditions are needed to find expressions for b and P_B It is assumed that the material is linearly elastic, homogenous and that displacements are small. To solve the problem two situations must be considered, the reference configuration and a time $t = t_1$ later at which time the external pressure has been altered.

2.1 Situation1: The Reference configuration

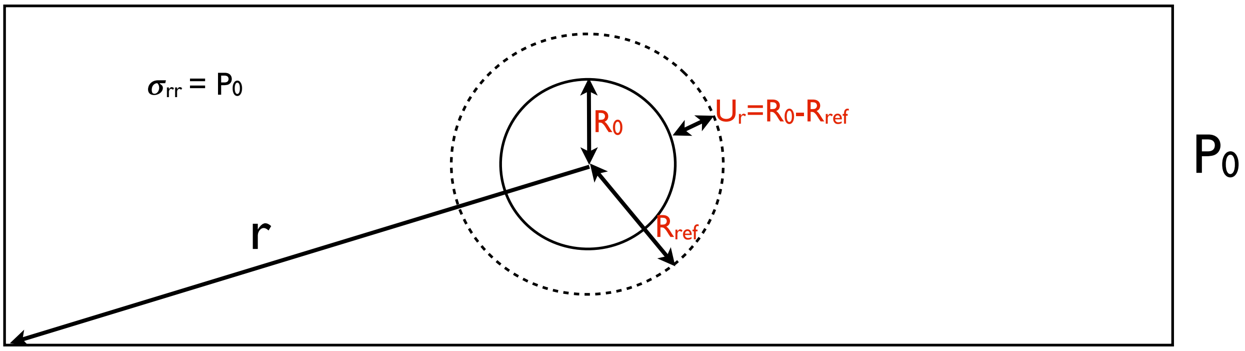

Supplementary Figure 1: Diagram showing the tissue containing a single bubble, the dotted line indicated the reference configuration i.e. a hypothetical "unstressed" configuration at which time there would be a bubble with radius R_{ref}. At time = 0 there is a pressure P_0 the radius is R_0 and the stress through the tissue is constant.

In this derivation we are considering an infinite block of tissue which at time 0 contains a bubble with radius R_0. As the external pressure changes the pressure at the outer boundary changes as does the stress at the bubble boundary. This causes a force imbalance at the bubble surface causing it to increase or decrease in radius until the pressure inside the bubble is once again in balance with the external pressure from the tissue. In this instance we are ignoring diffusion and just effectively looking at the boyle's law contribution to the bubble radius change as a consequence of change in external pressure.

Consider Supplementary Figure 1 Initially we have an initial external pressure of P_0, and an initial bubble radius or R_0. At this time there is a constant stress through the tissue of σ_{rr}

$$P_B = P_0 + \frac{2\gamma}{R_0} \tag{6}$$

$$\sigma_{rr} = -P_0 \tag{7}$$

Hence at the boundary i.e. when r=∞ (2) is only valid if:

$$b = 0 \tag{8}$$

and hence

$$a = -\frac{P_0}{3K} \tag{9}$$

So (2) becomes

$$U_r = -\frac{P_0 r}{3K} \tag{10}$$

For when $R_B = R_0$

At this point we use introduce the reference configuration. This is the configuration of the system

if it were unstressed. To do this we say that if σ_{rr} we zero throughout the tissue there would exist a void with a radius of R_{ref}. The displacement U_r will always be formulated as the difference between the current radius and this reference radius: $U_r = R_B - R_{ref}$ We already have an expression for this at $R = R_0$ give above. At later times we therefore have the displacement given as the displacement of R_0 plus the displacement between R_0 and the current R_B.

$$U_r = (R_B - R_0) - \frac{P_0 r}{3K} \tag{11}$$

From the above we also have the relation between R_0 and R_{ref} at $r = R_{ref}$ as:

$$R_0 = R_{ref} - \frac{P_0}{3K} R_{ref} \tag{12}$$

2.2 Situation 2: Expression for R_B

Consider Situation 2: If we now move to a new time point. At this time the external applied pressure P_{amb} has changed and the stress throughout the tissue is no longer constant. We have: At $r = R_{ref}$

$$\sigma_{rr} = -P_{int} \tag{13}$$

At $r = \infty$

$$\sigma_{rr} = -P_{amb} \tag{14}$$

where

$$-P_{amb} = P_B - \frac{2\gamma}{R_B} \tag{15}$$

$$3Ka = -P_{amb} \tag{16}$$

hence

$$a = -\frac{P_{amb}}{3K} \tag{17}$$

$$-P_{amb} - \frac{4b\mu}{R_{ref}^3} = -P_{int} \tag{18}$$

So we have four simultaneous equations from the above

$$-P_{amb} - \frac{4b\mu}{R_{ref}^3} = \frac{2\gamma}{R_B} - P_B \tag{19}$$

$$U_r = R_B - Rref = -\frac{P_{amb}}{3K} + \frac{b}{R_{ref}^2} \tag{20}$$

The ideal gas law gives us:

$$P_B R_B^3 = \left(P_0 + \frac{2\gamma}{R_0}\right) R_0^3 \tag{21}$$

$$R_0 = R_{ref}\left(1 - \frac{P_0}{3K}\right) \tag{22}$$

---

to summarise; the known and unknown quantities in the above 4 equations as

Know	Unknown
P_{amb}	R_{ref}
μ	R_B
P_0	P_B
R_0	b
K	
γ	

Now we want an expression for R_B in terms of the change in external pressure P_{amb} rearranging (22) we have

$$R_{ref} = \frac{R_0}{1 - \frac{P_0}{3K}} \tag{23}$$

Subbing (22) into (20)

$$R_B = \frac{\left(1 - \frac{P_{amb}}{3K}\right)}{\left(1 - \frac{P_0}{3K}\right)} R_0 + \frac{b}{R_0^2}\left(1 - \frac{P_0}{3K}\right)^2 \tag{24}$$

rearranging (21)

$$P_B = \left(P_0 + \frac{2\gamma}{R_0}\right)\left(\frac{R_0}{R_B}\right)^3 \tag{25}$$

subbing into (19)

$$P_B - \frac{2\gamma}{R_B} - P_{amb} = \frac{4b\mu}{R_{ref}^3} \tag{26}$$

rearranging for b:

$$b = \left(P_B - \frac{2\gamma}{R_B} - P_{amb}\right)\frac{R_{ref}^3}{4\mu} \tag{27}$$

Subbing in the expression for P_B and (22)

$$b = \left[\left(P_0 + \frac{2\gamma}{R_0}\right)\left(\frac{R_0}{R_B}\right)^3 - \frac{2\gamma}{R_B} - P_{amb}\right]\left(\frac{R_0}{1 - \frac{P_0}{3K}}\right)^3 \frac{1}{4\mu} \tag{28}$$

Substitute the expression for b into (24)

$$R_B = \left(\frac{1 - \frac{P_{amb}}{3K}}{\left(1 - \frac{P_0}{3K}\right)} R_0\right) + \left(\left(P_0 + \frac{2\gamma}{R_0}\right)\left(\frac{R_0}{R_B}\right)^3 - \frac{2\gamma}{R_B} - P_{amb}\right)\left(\frac{R_0}{4\mu\left(1 - \frac{P_0}{3K}\right)}\right)^3 \frac{\left(1 - \frac{P_0}{3K}\right)^2}{R_0^2} \tag{29}$$

simplifying we may obtain the quartic from which R_B can be found:

$$0 = R_B^4 + \left(\frac{P_{amb}}{4\mu} - \left(1 - \frac{P_{amb}}{3K}\right)\right)\frac{R_0}{\left(1 - \frac{P_0}{3K}\right)} R_B^3 + \frac{2\gamma R_0}{4\mu\left(1 - \frac{P_0}{3K}\right)} R_B^2 - \frac{R_0^4}{4\mu\left(1 - \frac{P_0}{3K}\right)}\left(P_0 + \frac{2\gamma}{R_0}\right) \tag{30}$$

Or by subsititution of (27) into (24) we can write the the expression in the form of the Young-Laplace equation:

$$P_B = P_{amb} + \frac{2\gamma}{R_B} + 4\mu\left(\left(1 - \frac{P_0}{3K}\right)\left(\frac{R_B}{R_0}\right) - \left(1 - \frac{P_{amb}}{3K}\right)\right) \tag{31}$$

3 Governing equations

Here we derive the governing equation for the in-silico model of bubble growth in a collagen gel tissue phantom.

During the compression and time at increased pressure, the tissue phantom will saturate with dissolved gas based on Ficks law of diffusion:

$$\frac{\partial C^g}{\partial t} = D \bigtriangledown^2 C^g \tag{32}$$

With the boundary conditions at the tissue edge given by Henry's Law:

$$C^g = k_h^g P_{amb}^g \tag{33}$$

Where C_g is the concentration of the gth dissolved gas, h_g is Henry's constant and P_{amb}^g is the partial pressure of the gth gas. All gases are considered to behave as ideal gases hence:

$$PV = n\mathcal{R}T \tag{34}$$

Where $\mathcal{R}$ is the gas constant, and T is the temperature. Alternatively

$$PV = \alpha_g m \tag{35}$$

where α is the specific gas constant, given by:

$$\alpha_g = \frac{\mathcal{R}T}{M} \tag{36}$$

where M is the molar mass of the gth gas. (Temp is assumed to be 37°).

During decompression the external pressure decreases and bubbles will grow by both Boyle's Law:

$$P_1V_1 = P_2V_2 \tag{37}$$

and by diffusion of dissolved gas into the bubble following Fick's first law:

$$\frac{dm_g}{dt} = 4\pi R_B^2 D \frac{\partial C_B^g}{\partial r}\bigg|_{R_B} \tag{38}$$

Where again Henry's Law is used as the boundary condition for (38):

$$C_B^g = k_h^g P_B^g \tag{39}$$

P_B^g is the internal pressure of the bubble and this is described by the Young-Laplace equation

$$P_B^g = P_{amb}^g + 2\gamma/R_B + \Omega(R_B) \tag{40}$$

Where $\Omega(R_B)$ is a term to account for the elastic resistance of the tissue to bubble growth. Substituting m in (38) with the perfect gas law we have

$$\frac{d}{dt}\left(\frac{P_B^g V_B}{\alpha_g}\right) = 4\pi R_B^2 D \frac{\partial C_B^g}{\partial r}\bigg|_{R_B} \tag{41}$$

Expanding the LHS of (41) with the product rule and including the separate affects of both N_2 and O_2 we have

$$\frac{d}{dt}\left(\left(\frac{P_B^{N_2}}{\alpha_{N_2}} + \frac{P_B^{O_2}}{\alpha_{O_2}}\right)V_B\right) = 4\pi R_B^2 D \frac{\partial (C_B^{N_2} + C_B^{O_2})}{\partial r}\bigg|_{R_B} \tag{42}$$

Assuming the mole fractions are constant throughout and using the substituting in the Young-Laplace equation to the LHS of (42) we find

$$\left(\frac{m_f^{N_2}}{\alpha_{N_2}} + \frac{m_f^{O_2}}{\alpha_{O_2}}\right)\left(P_B 4\pi R_B^2 \frac{dR_B}{dt} + \frac{4\pi R^3}{3}\left(\frac{dP_{amb}}{dt} + \left(-\frac{2\gamma}{R_B^2} + \frac{d\Omega}{dR_B}\right)\frac{dR_B}{dt}\right)\right) = 4\pi R_B^2 D \frac{\partial (c_B^{N_2} + c_B^{O_2})}{\partial r}\bigg|_{R_B} \tag{43}$$

Rearranging for dR/dt

$$\frac{dR_B}{dt} = \frac{\frac{\alpha_{O_2}\alpha_{N_2}}{m_f^{N_2}{}^2\alpha_{O_2} + m_f^{O_2}{}^2\alpha_{N_2}} D \frac{\partial C_{tot}}{\partial r}|_{R_B} - \frac{R_B}{3}\frac{dP_{amb}}{dt}}{P_{amb} - \frac{4\gamma}{3R_B} + \Omega\frac{\partial\Omega}{dR_B}} \tag{44}$$

Where C_{tot} is the sum of the individual gas contributions. These equations can be implemented for any number of gases. Current simulations use only Nitrogen and Oxygen. Where multiple gases are used the partial pressure of each gas is calculated from the mole fraction and the total pressure. This affects the boundary conditions and separate diffusion through the bulk is calculated for each gas.

4 Computational Implementation

To numerically implement the governing equations (2) and (5) the tissue phantom is defined as a 3-dimensional array of points with distance between each point. Each grid point or node is described by its 3D Cartesian co-ordinate and represents a unit volume of the system. At each node the concentration of dissolved gas and phase (liquid or gas) of the node is stored. The pressure profile is discretized into time portion . At each time point the pressure external to the tissue block P_{amb} is given by this profile. P_{amb} is used in Henrys Law (eq. 2) to set the tissue phantom dissolved gas boundary values at each time point. From these boundary values the dissolved gas concentration at every liquid node can be calculated. This provides a 3D dissolved gas concentration, which is then used to calculate the dissolved gas gradient at the tissue phantom bubble interface. This is used in Eq. (5) to calculate the new radius of the bubble. The governing equations are discretized a solved via finite difference methods. Diffusion through the bulk of the tissue phantom is solved to second order accuracy using a forward time centered space (FTSC) scheme, utilizing a seven point stencil. The pressure change and bubble radial change are discretized to first degree accuracy,

$$\frac{dP_{amb}}{dt} \approx \frac{P^{s}_{amb} - P^{s-1}_{amb}}{\delta t} \tag{45}$$

$$\frac{dR_B}{dt} \approx \frac{R^{s}_{B} - R^{s-1}_{B}}{\delta t} \tag{46}$$

4.1 Gas liquid interface

A system, such as the one described here, which consists of two phases, requires particular care in the treatment of the phase interface. The situation arises in many fields and there are two broad approaches: diffuse or sharp interface techniques. A diffuse interface technique assumes the phase boundary to have a finite thickness over which there is a smooth change in physical properties such as concentration. By comparison, sharp interface techniques model the boundary as a surface over which a discontinuous change in physical properties occurs. A diffuse interface technique requires a grid resolution of $h_i/4$ where hi is the interface thickness. This makes the technique less suitable for computational domains far larger than the interface thickness. One way to overcome this restrictions is the adaptive mesh schemes to refine the grid in the vicinity of the interface, however this technique requires computationally expensive re-gridding at each time step. In our case a sharp interface technique was employed on a fixed Cartesian mesh in this model, as high computational efficiency was deemed of greatest importance given both the size of the computational domain and the time scales over which simulations were required (several hours). Due to this approach specific methods for any computations involving grid points on or crossing either the phase boundary were required. Such computations occurred in two cases: i) during bulk diffusion calculations where one of the seven stencil points fell within the bubble (see Supplementary Figure 2). ii) For calculation of the concentration gradient at the bubble boundary.

In case i) the point in question is know as a boundary point, when a boundary point is reached in the calculation, the stencil point within the bubble is replaced by one at the bubble surface with concentration equal to CB. This caused the seven-point stencil to become asymmetrical and violate the stability criteria. Hence the dissolved gas concentration of the boundary point is found by inverse distance weighted interpolation from the surrounding neighbours rather than the FTSC scheme used for other bulk points. Case ii)- calculation of the gradient at the bubble surface $\left(\frac{\partial C^g_B}{\partial r}|_{R_B}\right)$ is dealt with using a normal probe method described in [2]. A set of spherically symmetrical points around each bubble is created as shown in Supplementary Figure 2 LHS. The concentration of these points is found using inverse distance weighting interpolation of the 6 nearest neighbours. Using the new array of spherical points a 1D forward difference approximation with 2nd order accuracy can be constructed in terms of the radial co-ordinate.

$$\frac{\partial C}{\partial R} = \frac{1}{2\delta r}\left(-C_{r+2} + 4C_{r+1} - 3C_B\right) \tag{47}$$

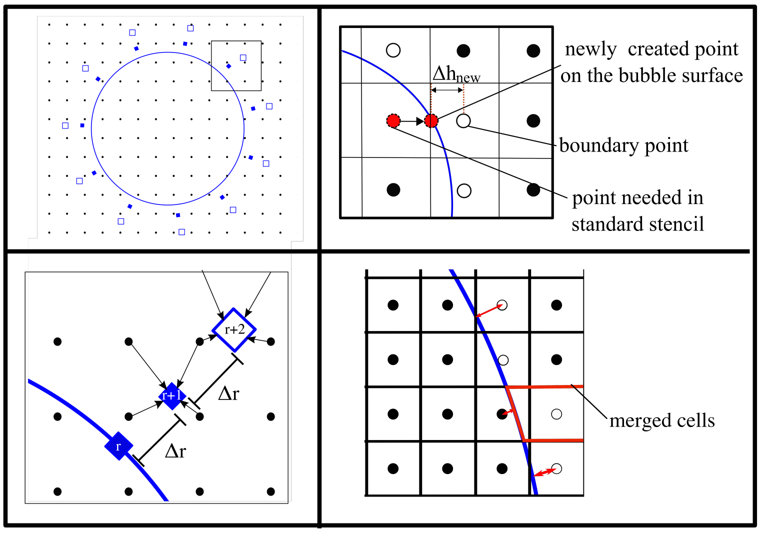

Supplementary Figure 2: Upper Left: 2D Schematic of the radial distribution of points surrounding the bubble within the Cartesian co-ordinate grid. Lower left inset from top left representing the calculation of the concentration for the radial points via interpolation from the surrounding Cartesian grid points. Upper right: 2D representation of the procedure for dealing with diffusion at a boundary point, the creation of a new stencil point in on the bubble boundary is clearly marked and the reduced grid spacing Δh new that violated the stability criteria. Lower right: 2D representation of the merging of grid cells at the bubble boundary to ensure local conservation of geometry and thus mass conservation.

where C_{r+2} and C_{r+1} are the concentrations at points $r+1$ and $r+2$, and r is the radial distance between points shown in Supplementary Figure 2. C_B is the concentration of gas at the bubble surface as given by Henrys law, in all simulations $\delta r = h$.

4.2 Mass Conservation

Mass conservation is of particular importance for sharp interface Cartesian grid methods. Such methods are prone to local violation of mass conservation due to the movement of the boundary over grid points, creating so called fresh and dead cells which may act as spurious sources and sinks. In addition errors may arise from the discrepancy between the actual smooth curved bubble surface and its computational depiction using the cuboid volume nodes (a so-call staircase error). In order to ensure mass conservation, the mass of gas in the bubble (as calculated from the perfect gas law) is compared to the total dissolved gas concentration of the tissue and the mass flux. Total mass of gas in the tissue must be calculated accounting for the partial volume of nodes which are cut by the bubble surface as shown in Supplementary Figure 2. If a mass deficit is found, the mass is transferred to the tissue nodes directly surrounding the bubble in proportion to their volume. This method can be shown to ensure good mass conservation by simulation of an oscillatory external pressure profile with no flux boundary conditions applied to the tissue edges (see Supplementary Figure 3).

5 Image Analysis

The error in bubble radius was established by measures of calibration standard metal parts, (measured with a micrometre), error in measurement of this was found to be 0.005mm. To analyse bubble growth profiles, time-lapse images of multiple bubbles within the tissue phantoms were taken starting at the commencement of decompression. The radial trajectories resulting from bubbles were fitted using a rhobust non-linear regression (Prism 6). The regression model chosen was

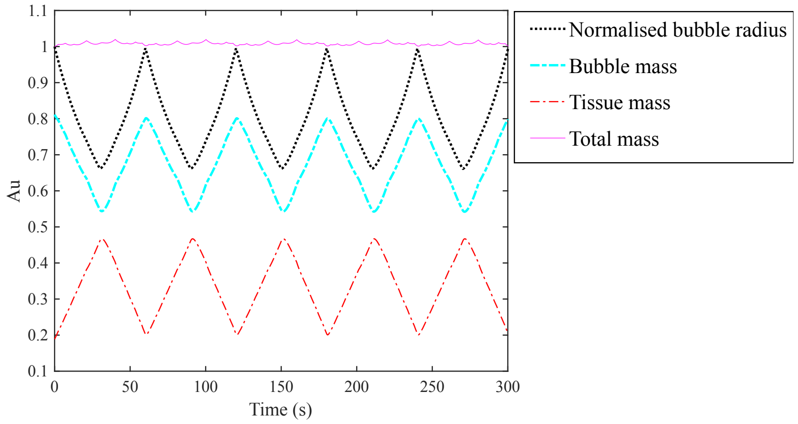

Supplementary Figure 3: Showing mass conservation for an oscillating bubble with no flux boundary conditions at the simulated tissue phantom edges.

a single-phase exponential decay equation described by:

$$R_B = (R_0 - Plateau) \cdot e^{-tln(2)/\tau} + Plateau \tag{48}$$

Plateau is the asymptotic value of the radius and τ is the half life of the bubble. The fit was constrained to ensure the plateau value was greater than the initial radius. The stages of image analysis and non-linear regression are shown in Supplementary Figure 4

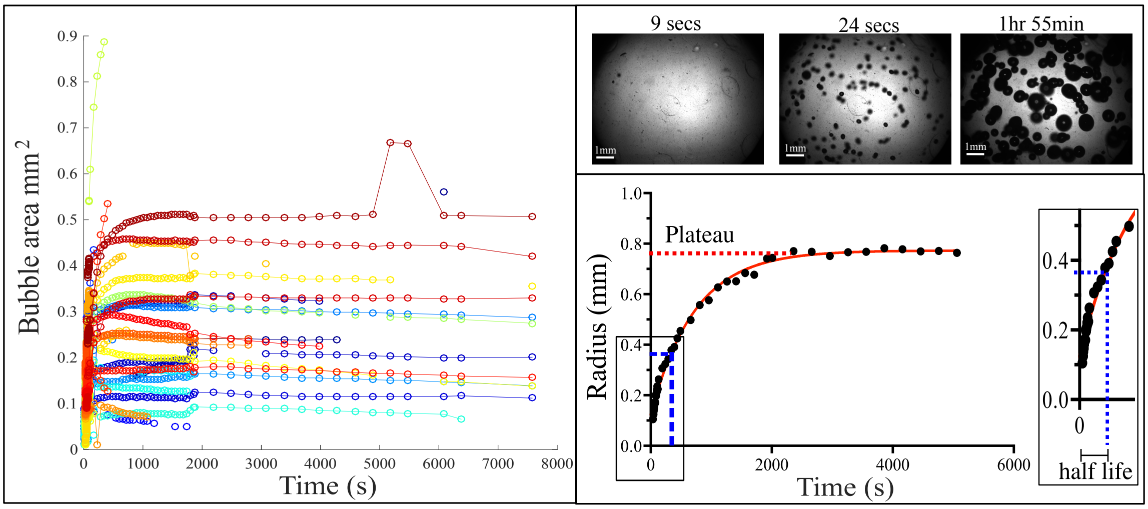

Supplementary Figure 4: Left hand panel showing a representative example of the experimental data extracted from the time lapse images. Each line represents a different bubble within the tissue phantom. Upper right: Representative examples of the time lapse images taken during a decompression. Lower right: Example of the non-linear fitting for a single bubbles complete radial trajectory, the rhobust regression is shown in red the Plateau radius is marked and the half-life shown in the inset.

6 Parameterisation

Material parameters were set based on a literature search, separate tables of the five material parameters are shown below. In addition the parameters α_{N_2} and α_{O_2} were taken from [3].

Conversion from Ostwald coefficient (L) to Henrys constant is done via the formula from

**Source**	**Value of** $D(m^2/s)$	**Material**	**Gas**	**Temp** $^\circ C$
[4]	4.5×10^{-10}	Collagen 11% w.t	O_2	37
[4]	1.7×10^{-10}	Collagen 34% w.t	O_2	37
[5]	1.88×10^{-9}	Water	N_2	25
[5]	2.10×10^{-9}	Water	N_2	25
[6]	1.7×10^{-9}	Water	O_2	37
[6]	$2.0 - 2.7 \times 10^{-9}$	Agar 2%	O_2	30
[7]	2.7×10^{-9}	Agar 2%	O_2	37
[8]	2.2×10^{-9}	cartilage	O_2	37
[9]	$7 \times 10^{-10} - 2 \times 10^{-9}$	Decellularized ECM	O_2	37
[10]	3×10^{-9}	Collagen (1mg/ml) 0.1%, (3mg/ml) 0.2%	O_2	30
[11]	8×10^{-10}	Aortic pig wall	N_2	37
[11]	2.9×10^{-10}	Plasma membrane cardeomyocyte	O_2	37
[12]	2.83×10^{-10}	Water	N_2	40
[12]	3.33×10^{-10}	Water	O_2	40

Table 1: Estimations of diffusion coefficients in various biological media

**Source**	**Value of** $k_h or L)$	**Material**	**Gas**	**Temp**
[11]	$0.0145(L)$	Blood Plasma	N_2	37
[11]	$0.027(L)$	Blood Plasma	O_2	37
[20]	$0.027(L)$	Water	O_2	37
[21]	$0.014520(L)$	Water	N_2	35
[11]	$0.073(L)$	Sheep bone marrow	N_2	37
[11]	$0.015(L)$	Calf Brain	N_2	37
[11]	$0.0133(L)$	Olive oil	O_2	37
[11]	$0.0261(L)$	Whole man	O_2	37

Table 2: Estimations of L in various biological tissues

Emerson [13] pg 87. (assuming the density of biological fluid to be 1), given by

$$L^g = \frac{k_h^g}{\mathcal{R}_{spec} T} \tag{49}$$

where $\mathcal{R}_{spec}$ is the specific gas constant for the gth gas. Values chosen for collagen gels were 0.027 for O2 and 0.0145 for N2, in the modelling literature there is also agreement with these values [14, 15, 16, 17, 18, 19].

Source	Value of γ mN/m	Liquid	Method	Temp
[22]	69.8	Water	Tensiometer	37
[22]	69.8	Saline	Tensiometer	37
[22]	46.4	Albumin	Tensiometer	37
[22]	40.5	Plasma	Tensiometer	37
[22]	40.6	Serum	Tensiometer	37
[23]	73	Saline	Microscopy	Not reported
[24]	73	Polyacrylamide hydrogel	spherical harmonic of liquid drops	Room temp

Table 3: Estimations of γ in various biological tissues

Source	Value of $\mu(Pa)$	Material	Method	Temp
[25]	$6.32 - 318$ (aggregate modulus)	1mg/ml Collagen	confined compression	37
[26]	$10 - 100$	0.9mg/ml Collagen	cone plate rheology	37
[27]	$0.2 - 100$	0.5mg/ml-1mg/ml Collagen	optical tweezers	21
[28]	$53 - 40$	2.3mg/ml-1.5mg/ml Collagen	optical tweezers	37
[29]	2	$2mg/ml$ Collagen	Bholin Gemini rheometry	Unspecified
[30]	15.5	2.1mg/ml Collagen	coquette rheometry	Unspecified
[31]	$300 - 350$	2.4mg/ml Collagen	Bholin CVO rheometry	37
[32]	23	4mg/ml Collagen	Plate rheology	37
[33]	$0.75k - 2k$	Liver	Various	*in vivo*
[34]	$0.173M - 0.4M$	Articulate cartilage	Various	*in vivo*
[35]	10.6 ± 5.3	Turkey breast	Elastography	Unspecified
[35]	10.3 ± 1.5	Bovine liver	Elastography	Unspecified
[35]	7.1 ± 2.1	Agar-Gelatin	Elastography	Unspecified
[35]	46.7 ± 28.7	Poricine Fat tissue	Elastography	Unspecified

Table 4: Estimations of μ in various biological soft tissues and tissue mimics

7 Bubble nucleation

Bubble nucleation is the subject of a separate publication however the distibution of nuclei was investigated through images of the tissue phantoms after a decompression profile by removing the phantoms from the pressure chamber and imaging them in a cross sectional view with phantoms placed in a clear petri dish containing PBS and a digital camera mounted to the side. The images were analysed manually in imageJ by taking a random rectangular slice of the whole depth of the phantom and marking the centre of all bubbles in the image section. The vertical distance of the bubble from the phantoms upper surface was normalised to the phantom thickness and plotted as a histogram and a cumulative frequency distribution shown in Supplementary Figure 5.

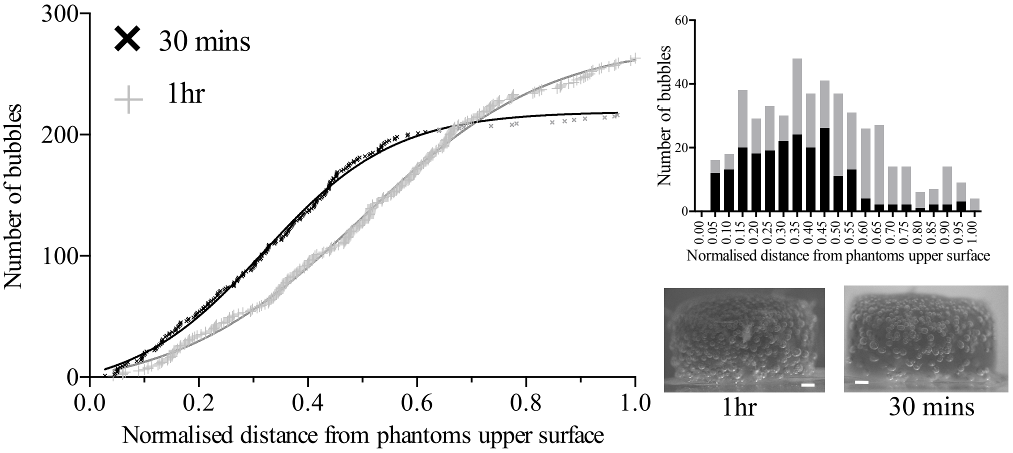

Supplementary Figure 5: Showing the distribution of bubbles in the z-plane. Right hand side shows the histogram of bubble z positions as measured from the top surface of the bubble and below representative images of the two cases (scale bar=1mm). Left hand side shows the cumulative frequency distribution for each decompression condition. A sigmoidal curve is fitted to both sets of data showing showing that the distributions are both non-uniform. Non-linear regression shows that the each distribution is distinct with the 1hr data being statistically closer a straight line (uniform distribution) than the 30min data. For both cases N=3.

References

[1] Landau LD, Lifshitz EM, Sykes JB, Reid WH, Dill EH. Theory of Elasticity: Vol. 7 of Course of Theoretical Physics; 1960.

[2] Udaykumar H, Mao L, Mittal R. A finite-volume sharp interface scheme for dendritic growth simulations: comparison with microscopic solvability theory. Numerical Heat Transfer: Part B: Fundamentals. 2002;42(5):389–409.

[3] The Individual and Universal Gas Constants;. Available from: http://www.engineeringtoolbox.com/individual-universal-gas-constant-d$_5$88.$html[cited07/01/2016]$.

[4] Cheema U, Rong Z, Kirresh O, Macrobert AJ, Vadgama P, Brown RA. Oxygen diffusion through collagen scaffolds at defined densities : implications for cell survival in tissue models. Tissue Engineering. 2012;6(1):77–84.

[5] Cussler EL. Diffusion: Mass Transfer in Fluid Systems. Cambridge Series in Chemical Engineering. Cambridge University Press; 2009. Available from: https://books.google.co.uk/books?id=dq6LdJyN8ScC.

[6] Chaix E, Guillaume C, Guillard V. Oxygen and Carbon Dioxide Solubility and Diffusivity in Solid Food Matrices: A Review of Past and Current Knowledge. Comprehensive Reviews in Food Science and Food Safety. 2014;13(3):261–286. Available from: http://doi.wiley.com/10.1111/1541-4337.12058.

[7] Vaupel P. Effect of percentual water content in tissues and liquids on the diffusion coefficients of O2, CO2, N2, and H2. Pflügers Archiv European Journal of Physiology. 1976;361(2):201–204.

[8] Malda J, Rouwkema J, Martens DE, le Comte EP, Kooy FK, Tramper J, et al. Oxygen gradients in tissue-engineered Pegt/Pbt cartilaginous constructs: Measurement and modeling. Biotechnology and Bioengineering. 2004;86(1):9–18. Available from: http://dx.doi.org/10.1002/bit.20038.

[9] Androjna C, Gatica JE, Belovich JM, Derwin KA. Oxygen diffusion through natural extracellular matrices: implications for estimating "critical thickness" values in tendon tissue engineering. Tissue Engineering Part A. 2008;14(4):559–569.

[10] Colom A, Galgoczy R, Almendros I, Xaubet A, Farré R, Alcaraz J. Oxygen diffusion and consumption in extracellular matrix gels: Implications for designing three-dimensional cultures. Journal of Biomedical Materials Research - Part A. 2014;102(8):2776–2784.

[11] Langø T, Mørland T, Brubakk A. Diffusion coefficients and solubility coefficients for gases in biological fluids and tissues: a review. Undersea & hyperbaric medicine: journal of the Undersea and Hyperbaric Medical Society, Inc. 1996;23(4):247–272.

[12] Ferrell RT, Himmelblau DM. Diffusion coefficients of nitrogen and oxygen in water. Journal of Chemical & Engineering Data. 1967;12(1):111–115. Available from: http://dx.doi.org/10.1021/je60032a036.

[13] Emerson S, Hedges J. Chemical Oceanography and the Marine Carbon Cycle. Cambridge University Press; 2008.

[14] Gernhardt ML. Development and evaluation of a decompression stress index based on tissue bubble dynamics. University of Pennsylvania; 1991.

[15] Burkard ME, Van Liew HD. Simulation of exchanges of multiple gases in bubbles in the body. Respiration physiology. 1994 Feb;95(2):131–45. Available from: http://www.ncbi.nlm.nih.gov/pubmed/8191036.

[16] Hugon J, Rostain JC, Gardette B. A new biophysical decompression model for estimating the risk of articular bends during and after decompression. Journal of theoretical biology. 2011 Aug;283(1):168–79. Available from: http://www.ncbi.nlm.nih.gov/pubmed/21609722.

[17] Gürmen NM, Llewellyn aJ, Gilbert Ra, Egi SM. Simulation of dynamic bubble spectra in tissues. IEEE transactions on bio-medical engineering. 2001 Feb;48(2):185–93. Available from: http://www.ncbi.nlm.nih.gov/pubmed/11296874.

[18] Srinivasan RS, Gerth Wa, Powell MR. Mathematical Model of Diffusion-Limited Evolution of Multiple Gas Bubbles in Tissue. Annals of Biomedical Engineering. 2003 Apr;31(4):471–481. Available from: http://link.springer.com/10.1114/1.1561288.

[19] Chappell MA, Payne SJ. A physiological model of the interaction between tissue bubbles and the formation of blood-borne bubbles under decompression. Physics in medicine and biology. 2006 May;51(9):2321–38. Available from: http://www.ncbi.nlm.nih.gov/pubmed/16625045.

[20] Rettich TR, Battino R, Wilhelm E. Solubility of gases in liquids. 22. High-precision determination of Henry's law constants of oxygen in liquid water fromT = 274 K toT = 328 K. The Journal of Chemical Thermodynamics. 2000;32(9):1145 – 1156. Available from: http://www.sciencedirect.com/science/article/pii/S0021961499905815.

[21] Rettich TR, Battino R, Wilhelm E. Solubility of gases in liquids. XVI. Henry's law coefficients for nitrogen in water at 5 to 50C. Journal of Solution Chemistry. 1984;13(5):335–348. Available from: http://dx.doi.org/10.1007/BF00645706.

[22] Mottaghy K, Hahn A. Interfacial tension of some biological fluids: a comparative study. Clinical Chemistry and Laboratory Medicine. 1981;19(5):267–272.

[23] Kharge AB, Wu Y, Perlman CE. Sulforhodamine B interacts with albumin to lower surface tension and protect against ventilation injury of flooded alveoli. Journal of Applied Physiology. 2015;118(3):355–364. Available from: http://jap.physiology.org/lookup/doi/10.1152/japplphysiol.00818.2014.

[24] Chakrabarti A, Chaudhury MK. Direct measurement of the surface tension of a soft elastic hydrogel: Exploration of elastocapillary instability in adhesion. Langmuir. 2013;29(23):6926–6935.

[25] Knapp DM, Barocas VH, Moon AG. Rheology of reconstituted type I collagen gel in confined compression. Journal of Rheology (1978-present). 1997;41(5):971–993.

[26] Hsu S, Jamieson A, Blackwell J. Viscoelastic studies of extracellular matrix interactions in a model native collagen gel system. Biorheology. 1993;31(1):21–36.

[27] Shayegan M, Forde NR. Microrheological characterization of collagen systems: from molecular solutions to fibrillar gels. PloS one. 2013 Jan;8(8):e70590.

[28] Velegol D, Lanni F. Cell traction forces on soft biomaterials. I. Microrheology of type I collagen gels. Biophysical journal. 2001 Sep;81(3):1786–92.

[29] Basu A. Shear defomation in polymer gels and dense colloidal suspensions. University of Pennsylvania; 2012.

[30] Barocas VH, Moon AG, Tranquillo RT. The fibroblast-populated collagen microsphere assay of cell traction force—Part 2: Measurement of the cell traction parameter. Journal of biomechanical engineering. 1995;117(2):161–170.

[31] Rheology (gel stiffness of an elastic material) of PureCol Collagen product. advanced biomatrix; 2013. Available from: http://www.advancedbiomatrix.com/wp-content/uploads/2013/07/PureCol-Collagen-Gel-Stiffness-and-Rheology-Resutls1.pdf.

[32] Raub CB, Suresh V, Krasieva T, Lyubovitsky J, Mih JD, Putnam AJ, et al. Noninvasive assessment of collagen gel microstructure and mechanics using multiphoton microscopy. Biophysical journal. 2007 Mar;92(6):2212–22.

[33] Palmeri ML, Wang MH, Dahl JJ, Frinkley KD, Nightingale KR. Quantifying Hepatic Shear Modulus In Vivo Using Acoustic Radiation Force. Ultrasound in Medicine and Biology. 2008;34(4):546–558.

[34] Mow VC, Guo XE. Mechano-electrochemical properties of articular cartilage: their inhomogeneities and anisotropies. Annual review of biomedical engineering. 2002;4:175–209.

[35] Glozman T, Azhari H. A Method for Characterization of Tissue Elastic Properties Combining Ultrasonic Computed Tomography With Elastography. Journal of Ultrasound in Medicine. 2010;29(3):387–398. Available from: http://www.jultrasoundmed.org/content/29/3/387.abstract.
